# Post-closure Cost Efficiency in Public Versus Private Landfills: The Case of Emilia-Romagna (Italy)

**DOI:** 10.1007/s00267-023-01809-w

**Published:** 2023-03-18

**Authors:** Mouhcine Tallaki, Enrico Bracci, Riccardo Ievoli

**Affiliations:** 1 Department of Economics & Management, University of Ferrara, Via Voltapaletto, 11 44121 Ferrara, Italy; 2 Department of Chemical, Pharmaceutical and Agricultural Sciences, University of Ferrara, Via Luigi Borsari, 46 44121 Ferrara, Italy

**Keywords:** Landfill management, Post-closure management, Efficiency, Public/private management, Value for money

## Abstract

Waste management systems have developed in recent years toward the adoption of sustainable management principles and practices, such as circular economy, zero waste, resource efficiency, waste avoidance, re-use, and recycling. Nevertheless, landfills continue to be used for waste disposal despite their risks related to contamination and effects on urban development. Most research on landfills focuses on their operational and technical aspects, while the performance and cost efficiency in managing landfills is less commonly studied, especially their post-closure management. However, improving efficiency is very relevant in the context of scarce public sector resources. This paper, therefore, analyzes the efficiency of post-closure management of landfills. Drawing on agency and stewardship theories, we focus on the difference in efficiency between public and private management of post-closure landfills. We use a linear mixed regression model to analyze data from 2015 to 2018 relating to 54 landfills (79% of which are privately managed) in the Emilia-Romagna region of Italy. The results show that public management is more efficient than private management. Results contribute to defining drivers of cost and confirming a disparity in the performance of private and public management. Our results cast doubt on the assumption, which is prevalent in new public management theory, that private operators are more efficient than public ones. We conclude by highlighting that to reach efficiency, it is better to increase the effectiveness of regulation in terms of value for money, without pre-determined preferences for the type of management.

## Introduction

Landfill disposal is one of the most common means of disposing of waste, due to its low cost technology, despite its environmental impact (Weng et al., 2015; Brennan et al., 2016). As reported by Eurostat, in EU countries, despite efforts to reduce landfills and encourage sustainable waste management, about 38.7% of total waste produced was sent to landfills and about 38.1% was recycled in 2018. The performance and cost efficiency of landfills have been studied in relation to operational, managerial, and organizational issues. Previous research on landfills focused on various operational aspects, such as recovery of material from landfills (Wang et al., 2021), groundwater contamination management (Yang et al., 2016), risks of land re-use (Nai et al., 2021) and restoration (Kim and Lee, 2005), optimal localization of landfills (Franco et al., 2021), landfill mining (Jones et al., 2013; Danthurebandara et al., 2015), gas emissions in landfills (Nevrlý et al., 2019), the settlement of landfills (Jiangying et al., 2004), lining practices and leak detection methods (Pandey and Shukla, 2019), and leachate generation and reduction (Bilgili et al., 2006). Little or no research has been devoted so far to the role of management types in post-closure landfill management (Nguyen et al. 2021). The post-closure management of landfills represents the most important phase in terms of financial and environmental performance, as it can last from 30 to 50 years (Weng et al., 2015). Landfills can be managed by a public or private entity or a public–private partnership (Bel and Costas, 2006; Hefetz and Warner, 2007). The involvement of the private sector in waste management through privatization/liberalization was promoted by new public management (NPM) theory, which emphasized market structure and incentives as drivers for performance (Bel and Warner, 2008a). To test the assumption that privatization leads to increased efficiency, in this paper, we analyze the differences between privately and publicly owned organizations in managing post-closure landfills, in terms of efficiency.

In doing so, we draw on agency and stewardship theories, showing the difference in efficiency between public and private management of post-closure landfills. We use a linear mixed regression model to analyze data from 2015 to 2018 relating to 54 landfills in the Emilia-Romagna region of Italy.

The results contribute to this field by casting doubt on the assumption, which is prevalent in NPM, that private operators are more efficient than public ones. We conclude by highlighting that to achieve efficiency, it is better to increase the effectiveness of the regulatory framework in terms of value for money, without pre-determined preferences for the type of management.

## Landfill Cost Efficiency: Focus on the Type of Management and Ownership

Landfill efficiency depends on various factors, with the literature focusing mainly on operational characteristics. For instance, energy production, use, and capacity of landfills are some of the characteristics considered to impact efficiency (Niskanen et al., 2013; Kale and Gökçek, 2020). Specifically, energy recovery and energy production could reduce costs and boost performance (Behrooznia et al. 2018; Kale and Gökçek, 2020). Leachate composition (Camba et al., 2014) and landfill technologies (Nai et al., 2021; Wang et al., 2021) also affect efficiency. Pandey and Shukla (2019) highlighted that landfills, in particular old ones, lack adequate modern environmental technology, which leads to consequences in terms of costs. Berge et al. (2009) found that traditional landfills as well aerobic bioreactor landfills are less expensive than bioreactor landfills. Other factors that affect the performance and efficiency of landfills are localization and energy recovery potential. Franco et al. (2021) used the concepts of the facilities location problem to define the best ways to locate landfills and reduce the cost of transport and management. Performance of landfill management could also depend on urban population density, quantity and quality of waste, income, public support, political membership, extent of social benefits, and GDP per capita (Benito-López et al., 2011; Jacobsen et al., 2013). In this sense, country context is relevant to the performance of waste management (i.e., landfill management). In fact, different levels of performance have been achieved in developed and developing countries (Ferronato et al., 2019).

The efficiency and performance of landfill management may also depend on organizational factors, such as the type of ownership (public or private), management (public or private), and good governance (Bel and Warner, 2008b; Benito et al., 2010). Various researchers have reported the benefits of the privatization of public services (Bel and Warner, 2008a; Benito et al., 2010). Indeed, one reason commonly used to legitimize the participation of private operators in waste management, including landfill management, is that private operators are more efficient (Bel and Warner, 2008a; Benito et al., 2010) due to their managerial competencies (Berenyi and Stevens, 2013). However, there is no consensus regarding the benefit of private over public management. Alfiero et al. (2017) concluded that the involvement of private operators in the ownership of landfills enhances the performance of waste management. Others highlighted that evidence of cost savings in waste management is somewhat ambiguous (Bel and Costas, 2006; Bel and Warner, 2008b). In fact, Bel and Warner (2008b) emphasized there is little support for assumptions about the relationship between privatization and cost savings. Ownership and type of management, in this sense, are not relevant to explaining cost differences and performance in managing waste services. According to Bel and Warner (2008b), the key to efficiency in managing waste services is to increase competition and the number of bidders and to decrease monopoly and concentration in waste management. Competition encourages cost reductions and increases efficiency (Bel and Costas, 2006). Others have argued that sector regulation is the best way to achieve cost efficiency in managing waste as regulation can replace competition (Marques and Simões, 2008; Simões et al., 2010). Moreover, even if there is a potential benefit to privatization, in any case, this decreases over time as competition declines (Dijkgraaf and Gradus, 2007, 2008). The persistence of benefit depends on the trade-off between possible market failures due to a lack of competition and deficiencies in government control of public organizations. Therefore, competition and effective regulation seem to be more important than ownership per se (Yarrow et al., 1986).

There are conflicting results regarding the cost efficiency of public and private waste management (Bel and Costas, 2006; Bel and Warner, 2008b). Debate about the efficiency of private organizations with respect to public ones is ongoing (Alfiero et al., 2017). On the one hand, supporters of NPM hypothesize the benefit of adopting a market-based approach in the management of waste services. On the other hand, the empirical results are contradictory and do not entirely agree on the benefit of private sector involvement. Considering the scarcity of public sector resources, improving efficiency of landfill management is of great importance, with the alternative being increased costs for citizens. Increased costs lead to risks for the legitimacy of elected politicians, who may also struggle to find additional resources. So, improving efficiency remains the best option (Guerrini et al., 2017).

## Landfills and Cost Efficiency: Theoretical Development

Public versus private dualism has become central in the 80 s since the introduction of NPM and the associated reforms of outsourcing and privatization of public services. Privatization challenged, in terms of service provision, the traditional thinking that had been prevalent in public organizations. NPM represented a trend toward changing management style by promoting competition and the marketization of public services provision. NPM advocated an innovative managerial approach for public sector organizations, which were characterized by high bureaucracy and feeble organizational commitment (Boyne, 2002). In this sense, NPM promotes greater efficiency and effectiveness in managing public services (Massoud et al., 2002) to increase value for money, which is the basis for outsourcing and privatization of public services (Grimsey and Lewis, 2005). Therefore, NPM accelerated privatization and the outsourcing agenda of public services (Hood, 1991, 1995), including waste services. Hood (1991) stated that NPM “*focused the shift on privatization and away from core government institutions, with renewed emphasis on ‘subsidiarity’ in Service provision*.” In this context, public sector organizations define policies and set standards while the private entity acts within the contractual and regulatory framework (Kelly, 1998). Contracts and regulations are introduced to balance private and public interests. Private entities strive to maximize profits and return on capital invested; public sector organizations aim to guarantee the quality of the service and reduce the costs for citizens (O’Flynn, 2007; Bel and Warner, 2008a).

This has mobilized various theories to justify the choice of privatization, on the one hand, and the relationship between the public and private sectors, on the other. Agency theory, stewardship theory, and public choice theory are most prevalent (Davis et al., 1997; O’Flynn, 2007; Schillemans and Bjurstrøm, 2020). Public choice theory and agency theory are most commonly indicated for the analysis of public–private dualism in the context of privatization. Public choice theory recognizes the inefficiency of the public operator and pushes policy makers to adopt structures that improve efficiency. This theory was the basis for the separation and fragmentation that led to the privatization of various services. Public choice theory recognizes, therefore, the efficiency of the private operator. Agency theory focuses on the relationship between private and public operators considering that they could have divergent interests. The main challenge, according to this theory, is to provide a structure capable of creating incentives to align the interests of the parties involved (Foss, 1995).

When we talk about aligned interests between ownership and managers, we refer to stewardship theory. This theory highlights the collaborative and trustworthy characteristics of individuals and, contrary to agency theory, situations in which managers/subordinates are stewards whose objectives align with those of the principal agent (Davis et al., 1997).

In this paper, we draw on both agency and stewardship theories. Agency theory assumes that privatization reforms are built around the relationship between a principal (the public) and an agent (the private) (O’Flynn, 2007). In fact, as specified by Kelly (1998), the public policy maker “*articulates the policy, sets performance standards, and chooses in a competitive market an agent who will faithfully act in the government’s behalf to deliver the goods and services so that the outcome sought will be attained.”* Agency theory considers subordinates/managers as opportunistic and individualistic and depicts them as agents with divergent interests from the principal (the owner). In this case, individuals look at and try to maximize their utility and profit. According to agency theory, the private operator (agent), considering information asymmetry, individualism, and opportunism, tries to maximize profits. This occurs especially in a contractual relationship and where an agent’s actions could impact the well-being of the parties (Foss, 1995; Petersen, 1995). In this case, an incentives control structure should align the interests of the parties involved (Foss, 1995).

After the construction period, which is relatively short, landfills enter the management period, when operators dispose of waste in landfills for a fee per ton. When the landfill’s maximum capacity is reached, the post-closure period begins (which is relatively longer than the construction and use periods). Operators are obliged to maintain the landfill until the end of the closure period. Part of the disposal fees collected during the use period is set aside to provide for post-closure costs. If these funds are insufficient, additional public contributions or levies may be needed. So, these funds are quantified ex ante, and they are the key to the long-term satisfactory operation of the landfill (Lee and Jones-Lee, 2004). In the context analyzed in this paper, the economic control of landfills is entrusted to the Local Agency for Water and Waste Services of the Emilia-Romagna Region (ATERSIR). The control method established by law provides for an ex-post reporting of the costs incurred to ensure post-mortem maintenance. No incentives are foreseen for operators to reduce management costs, which remains one of the main objectives of the regional authority. If the fund is insufficient, the costs are charged to the tariff paid by the citizen (resolution no. 1441/2013, Emilia-Romagna region). The control structure does not incentivize efficiency. Private managers could adopt opportunistic behavior aimed at shifting part of the costs of other management to the landfill. So, the availability of these funds and the control structure adopted, from an agency theory perspective, could be a source of opportunistic behavior from private operators. Consequently, private management may result in inefficiency due to agency cost, which arises due to conflicts of interest and lack of incentives to align them. Organizational structure control and financial incentives could be used to reduce information asymmetry and to align the goals of the principal with those of the agent, thereby stemming agency costs (Jensen and Meckling, 1976; Davis et al., 1997).

However, when the interests of the owner and management of the landfill align, we cannot talk about the agent and principal. In such cases, we draw on stewardship theory, which is a useful framework to argue that an organization performs well when the interests of ownership and management coincide. Accordingly, publicly owned organizations may perform better than private ones. Stewardship theory, in opposition and complementary to agency theory, considers that managers have an incentive to make decisions in line with their organization, as the benefit of the organization indirectly becomes that of the actors belonging to the organization (Davis et al., 1997). Considering the above, our research question is as follows:

RQ1: Are landfills managed by private operators less or more efficient than those managed by public sector operators?

## Research Context and Methodology

Waste management in Italy involves the central state, regions, and municipalities (Decree Law n. 152/2006). The central state provides guidance, coordinates services, and defines the general criteria and methods for integrated waste management. The main functions of the regions consist in the preparation, adoption, and updating of regional waste management plans. In addition, regions regulate and control waste management activities. The municipalities are indirectly involved in the regulatory/control process that aims to prevent, recover, collect, and treat waste. Management of landfills is regulated by regional decree n. 1441/2013. From an integrated perspective, the sustainability of the waste management system in Italy was analyzed by Di Maria et al. (2018) using quantitative methods and composite indicators. In terms of waste management, the relevance of the Emilia-Romagna region in the Italian context emerges from a set of contributions published in the previous decade. Among them, Passarini et al. (2011) focused on waste management indicators (waste generation and separate collection rate) at a territorial level, while Magrini et al. (2021) analyzed the evolution of urban waste management systems from 2008 to 2018, considering both environmental and economic indicators but also the institutional changes during the decade. Then, Magrini et al. (2022) presented a methodology to provide a societal life cycle costing approach, identifying negative externality in the transportation of waste. One of the peculiarities of Emilia-Romagna is the presence of a centralized entity, i.e., the ATERSIR, which is a regional agency that regulates local environmental public services. The disposal fee is approved each year by ATERSIR. There are ten operative landfills in the region^1^ (reference year: 2020), but only five dispose of urban waste.

### Methodology

To analyze the cost efficiency of post-closure landfill management, we examined primary sources in the form of landfill reports collected by ATERSIR for the period from 2015 to 2018, with a temporal window of *T* = 4. Data reported by the various operators were verified by ATERSIR. We had full access to all reports received from the various operators during the period. The variables in the reports include total cost items; leachate produced; landfill volume; years of activity; years after closure; public or private management; public or mixed property; connection to sewer; post-operative management fund; province; and municipality. The number of considered landfills is equal to 54. These represent the total of landfills in Emilia-Romagna (central-north region; in Italy, there are 20 regions). Therefore, the total number of observations should have been equal to *N* = n*T = 216. However, while there was data available for most landfills (42; 78%) for the whole period (2015 to 2018), in four cases (7%), data for only three consecutive years were available. For the remaining 8 (15%) landfills, only the last year was available. To analyze the data, we employed the linear mixed regression model, which is indicated for multilevel/hierarchical and longitudinal data (Bates et al., 2015). Data were analyzed using R Studio (R Core Team, 2016).

Considering the *i* landfill in the *t* year, the target variable total cost was obtained as follows:

$$TC_{i,t} = C_{Mon;i,t} + C_{ML;i,t} + C_{MBio;i,t} + C_{Main;i,t} + C_{PO;i,t} + C_{Oth;i,t},$$

where *TC* stands for the total cost, *C*_*Mon*_ is the monitoring cost, *C*_*ML*_ represents the management cost of leachate, the cost of biogas management is depicted through *C*_*MBio*_, *C*_*Main*_ stands for maintenance cost, *C*_*PO*_ is the cost due to post-operative management, and other costs are depicted through *C*_*Oth*_. All these components are greater than or equal to zero.

Considering the overall sample, the cost of leachate represents, on average, 50% of the total cost of a closed landfill in Emilia-Romagna, followed by the cost of maintenance (18%), other costs (17%), and monitoring costs (11%). Post-operative and biogas management play a less important role in the composition of the total cost (1.1% and 3.2%, respectively).

In the model constructed, the dependent variable (total cost) has been assimilated to the natural logarithm of total cost (log_c), which is here considered as a measure of the efficiency of post-closure landfills. The logarithm transformation is applied to smooth the distribution of total costs, which can be affected by (positive) extreme values. This transformation helps to improve the linear relationship with the selected covariates and allows a straightforward interpretation of the coefficients in terms of elasticity (Panzone et al., 2021). In studies regarding waste, the logarithm of total cost was also used by Greco et al. (2015) to identify the drivers of costs of solid waste collection in Italian municipalities. Fernández-Aracil et al. (2018) employed the logarithm of total cost to verify the factors related to waste collection service in Spain. Additionally, Greco et al. (2018) used the logarithm of total cost to quantify the impact of tourism on municipal waste costs in Italy. Furthermore, Honma and Hu (2021) employed the logarithm of costs and applied stochastic cost frontier analysis to Japanese municipalities.

## Model description

The independent variables used to explain the logarithm of the total cost are as follows:

✓ *log_leachate*: logarithm of quantity of leachate (in tons), available only for landfills with leachate greater than 0; leachate is a liquid mainly generated by precipitation percolating through waste
✓ *log_volume*: natural logarithm of the landfill volume (in m^3^)
✓ *Quantity_of_waste*: amount of waste (in tons) in a landfill divided by 100,000
✓ *Urban_waste*: percentage of municipal solid waste in a landfill; the definition of urban waste in Italy was established by Decree Law 116/2020 and includes six types of waste (e.g., domestic unsorted and separated waste) but also the waste generated by public maintenance
✓ *Years_of_activity*: number of years of activity before the closure of the landfill
✓ *Years_aft_closure*: number of years after the closure of the landfill
✓ *Management*: a dummy variable assuming values of “public” or “private”
✓ *Property*: a dummy variable assuming values of “public” or “mixed”
✓ *Sewer*: a dummy variable assuming values of 1 or 0 whether or not the landfill is connected to the sewer
✓ *Post_op*: a dummy variable assuming a value of 1 if a post-operative management fund exists for the landfill; otherwise, this variable assumes a 0 value
✓ *Years_aft_closure*∧*Post_op*: interaction between number of years after closure and post-operative management fund.

Together with the main independent variable (i.e., management), we also included and tested other variables, which have emerged in the literature as determinants of cost efficiency. Among previously published contributions, Lombrano (2009) studied the relationship between private and public management and costs by considering a sample of Italian regions, while Neto et al. (2009) proposed a model for sanitary landfill costs in Brazil. Greco et al. (2015) used private management as a predictor of municipality costs and found systematic evidence that private (collection) is associated with lower costs. Laner et al. (2012) discussed post-operative (aftercare) funds in a review article regarding closed landfills. Damgaard et al. (2011) and Brennan et al. (2016) pointed out the impact of treating leachate on total costs and also considered different landfill configurations. Other variables highlighted in the literature that could impact landfill cost efficiency, such as urban population density, quantity and quality of waste, income, public support, political membership, extent of social benefits, and GDP per capita (Bel and Costas, 2006; Guerrini et al., 2017), are not used in our model. The units of analysis are all located in a homogeneous area; therefore, these variables are not significant to our research.

The selected linear mixed regression model (Bates et al. 2015) is described through the following assumptions:

$$\left( {{{{\mathrm{Y}}}}\left| {{{\mathrm{B}}}} \right. = {{{\boldsymbol{b}}}}} \right) \sim N\left( {{{{\boldsymbol{X}}}}{\boldsymbol{\beta}} + {{{\boldsymbol{Zb}}}} + \varepsilon ;\sigma ^2{{{\boldsymbol{W}}}}^{ - 1}} \right);\,{{{\mathrm{B}}}} \sim N\left( {0,{{{\mathbf{\Sigma }}}}} \right),$$

where the variance-covariance matrix Σ should be positive semidefinite, Y is the outcome variable of interest (*log_c*), X is an *N* × *p* matrix storing the covariates previously discussed (i.e., *log_leachate, log_volume, Quantity_of_waste, Urban_waste, Years_of_activity, Years_aft_closure, Management, Property, Sewer, Post_op, Years_aft_closure*∧*Post_op*), and ***β*** = {*β*_1_*,…,β*_*p*_} is the vector containing the p (equal to 11) coefficients associated with these covariates, while *b* is a vector containing the coefficient of random intercepts collected in ***Z***. Furthermore, ***W***^−1^ is a matrix of known weights, ***ε*** stores the offset terms, and σ is a scale parameter. In this framework, the unconditional distribution of Y, i.e., the vector-valued random outcome variable, is ${{{\mathrm{Y}}}} \sim N\left( {{{{\mathbf{X}}}}{\boldsymbol{\beta}} + \varepsilon ;\sigma ^2{{{\mathbf{W}}}}^{ - 1}} \right)$. This model is applied in other contributions regarding waste management at the territorial level. Mazzanti et al. (2010) and Carvalho et al. (2015) applied the random effect model to model the municipal solid waste in Italian landfills and the efficiency of municipal waste services in New South Wales, respectively. From a slightly different perspective, Di Foggia and Beccarello (2020) employed regression models (considering a two-year period) to identify the drivers of municipal solid waste management with a sample composed of most Italian municipalities. Finally, Gibbons et al. (2014) used linear mixed models in the case of closed landfills to study the degradation of leachate constituent.

As a benchmark, we also consider a conventional linear regression:

$$Y = {{{\boldsymbol{X}}}}{\boldsymbol{\beta}} + {{{\boldsymbol{u}}}},$$

where the coefficients of vector ***β*** are estimated through ordinary least squares (OLS) and ***u*** is a vector of identically distributed innovations.

To mitigate the effect of the landfills with only one year of available data, we developed two models. In the second model, we excluded landfills with one year’s data. The estimation was carried out using R Studio (R Core Team, 2016) with the package Ime4 (Bates et al., 2015), which allowed us to apply the restricted maximum likelihood (REML) estimator of the parameters. P-values and confidence intervals for the random effect coefficients were obtained through the ImerTest package (Kuznetsova et al., 2017). To relax the hypothesis of normality in random effect models, we also applied the Swamy-Arora estimator (Swamy and Arora, 1972) through the plm statistical package (see Croissant and Millo, 2008).

## Results

### Descriptive analysis

Descriptive statistics are depicted in Table 1, divided by each year of analysis. Quantitative variables are summarized through mean ± standard deviation, while qualitative variables are presented through frequencies and percentages (in brackets). The distribution of the logarithm of landfill volume (log_volume) is very similar in terms of the years. The main reason for this is that the log_volume is essentially constant over time for each landfill. Regarding the quantity of waste, a decrease of descriptive mean can be observed in the last available year compared to the period from 2015 to 2017, due to the inclusion of 8 new landfills in the sample, presenting a smaller amount of waste tons. The percentage of solid urban waste in the considered landfills is around 75%, with negligible changes throughout the period. The closed landfills are evenly distributed between mixed and public property.

**Table 1:** Descriptive statistics of the variables

Variable	Overall (188)	2015 (44)	2016 (45)	2017 (46)	2018 (54)
*log_c (log (€))*	12.21 ± 1.25	12.45 ± 1.33	12.05 ± 1.24	12.01 ± 1.22	12.32 ± 1.18
*log_leachate (log (ton))*	8.22 ± 1.55	8.52 ± 1.54	8.22 ± 1.60	7.83 ± 1.51	8.29 ± 1.52
*log_volume (log (m*^*3*^*))*	13.04 ± 1.14	13.06 ± 1.16	13.05 ± 1.15	13.05 ± 1.14	13.01 ± 1.14
*Quantity_of_waste*	843569.2 ± 1117666	898367.5 ± 1226366	897655.5 ± 1213737	897683.6 ± 1201779	705186.7 ± 851476.8
*Urban_waste*	74.58 ± 25.03	74.82 ± 25.14	74.26 ± 25.13	74.45 ± 24.88	74.77 ± 25.69
*Years_of_activity*	14.81 ± 10.25	14.52 ± 9.80	15.07 ± 10.53	15.02 ± 14.64	14.64 ± 10.35
*Years_aft_closure*	14.47 ± 8.14	13.3 ± 7.88	14.36 ± 7.89	15.39 ± 7.85	14.74 ± 8.87
Management:					
*Public*	36 (19.1)	7 (15.9)	9 (20.0)	9 (19.6)	11 (20.8)
*Private*	152 (80.9)	37 (84.1)	36 (80.0)	37 (80.4)	42 (79.2)
*Property:*					
*Public*	102 (54.3)	24 (54.5)	25 (55.6)	26 (56.5)	27 (50.9)
*Mixed*	86 (45.7)	20 (45.5)	20 (44.4)	20 (43.5)	26 (49.1)
*Sewer*					
*Yes*	42 (22.3)	9 (20.5)	9 (20.0)	11 (23.9)	13 (24.5)
*No*	146 (77.7)	35 (79.5)	36 (80.0)	35 (76.1)	40 (75.5)
Post_op					
*Yes*	89 (47.3)	22 (50.0)	18 (40.0)	19 (41.3)	30 (56.6)
*No*	99 (52.7)	22 (50.0)	27 (60.0)	27 (58.7)	23 (43.4)
*Yrs_closure∧Post_op*					
*Post_op: Yes*	11.3 ± 8.8	10.0 ± 8.3	11.2 ± 9.0	12.5 ± 8.9	11.6 ± 9.2
*Post_op: No*	17.3 ± 6.3	16.5 ± 6.0	16.4 ± 6.4	17.4 ± 6.4	18.8 ± 6.6

Based on a preliminary bivariate analysis (simple linear regression), a linear relationship between the quantity of leachate and total costs of landfills emerges from the actual data. According to the Pearson’s correlation coefficient, there is a positive (and statistically significant) correlation between these two quantities (0.41, R^2^ equal to 0.17), while the logarithmic transformation is useful to improve this linear relationship (0.73, R^2^ equal to 0.53). Moreover, the correlation between these two variables (measured through Pearson’s correlation coefficient) decreases throughout the considered period, from 0.82 in 2015 to 0.58 in 2018 (even considering the logarithmic transformation). This fact also led to the presence of other possible determinants that should have a relevant impact on the costs.

### Linear Mixed Model Regressions

The presence of multicollinearity was checked in all the model specifications through the variance inflation factor (VIF). In particular, the VIF was always lower than 8 in the OLS models and lower than 7 in the linear mixed regression models. Thus, there is clear evidence against the presence of multicollinearity in the considered models.

Table 2 contains the results of linear mixed models in terms of estimates, standard errors (SE), and statistical significances (*p*-values associated with the t-tests). Model I is estimated through *N* = 188 observations, while Model II is estimated based on *N* = 180 observations, to mitigate the effect due to the presence of landfills with only one year of available data. Model II shows a slightly lower individual random effect coefficient.

**Table 2:** Results of linear mixed models

	*Model I* (*N* = 188)		*Model II* (*N* = 180)	
Fixed Effects	Estimate (SE)	t-test (signific.)	Estimate (SE)	t-test (signific.)
*Intercept*	6.694 (1.085)	***	5.251 (1.031)	***
*log_leachate*	0.479 (0.056)	***	0.490 (0.052)	***
*log_volume*	0.186 (0.099)	*	0.302 (0.093)	**
*Quantity_of_waste*	0.003 (0.009)		−0.005 (0.008)	
*Urban_waste*	−0.369 (0.339)		−0.658 (0.324)	*
*Years_aft_closure*	−0.032 (0.014)	*	−0.027 (0.013)	*
*Years_of_activity*	−0.005 (0.009)		−0.003 (0.008)	
*Management: Public*	−0.472 (0.196)	*	−0.484 (0.176)	**
*Property: Public*	0.113 (0.177)		0.202 (0.159)	
*Connection to sewer*	−0.712 (0.186)	***	−0.725 (0.179)	***
*Post_op*	0.511 (0.287)	°	0.557 (0.278)	*
*Yrs_aft_clos∧Post_op*	−0.022 (0.017)		−0.028 (0.016)	°
Random Effects				
*Landfills*	0.165 (0.406)		0.114 (0.338)	
*Residuals*	0.253 (0.503)		0.238 (0.488)	

Significance symbols: *p*-value < 0.1 (°); *p*-value < 0.05 (*); *p*-value < 0.01 (**); *p*-value < 0.001 (***)

According to Model I, variables such as logarithm of leachate, logarithm of volume, years of activity, connection to the sewer, and public management are the main determinants of the total costs (in the logarithm), considering a nominal level of 0.05. For Model II, percentage of urban waste and post-operative fund are also statistically significant at a nominal level of 0.05.

To summarize the main results, the logarithm of leachate, logarithm of volume, and presence of post-operative fund are associated with higher costs (in the logarithm); percentage of urban waste, years after the closure, public management, and connection to the sewer are significant variables for cost reduction.

In particular, for each additional point of logarithm of leachate, the logarithm of the total costs increases (on average) 0.48 in Model I and 0.49 in Model II. Furthermore, for a unitary increase of landfill volume, the logarithm of total cost increases (on average) 0.186 according to Model I (the estimated coefficient is 0.302 for Model II); the length of time in years from the closure determines, on average, a decrease of about 0.03 in the logarithm of costs per year in Models I and II. The presence of a connection to the sewer in the landfill leads to an average decrease of costs (in the logarithm) of about 0.71 in Model I and 0.73 in Model II. Landfills with public management have (on average) lower logarithm of total costs in both models, with an estimated coefficient equal to −0.47 (Model I) and −0.48 (Model II). According to Model II, each additional percentage point of urban waste leads to a decrease in the costs (in the logarithm) of about 0.66; landfills with post-operative funds present, on average, increased costs of about 0.56. Taking a greater nominal level (0.1), the presence of a post-operative fund can also increase the costs (in the logarithm) of the landfill by 0.511 in Model I. Considering the same nominal level, Model II shows a weak significance of the interaction effect between post-operative fund and years after closure, with a negative impact on the total cost. To conclude, the results obtained through the Swamy-Arora estimator (presented in the Appendix) are very similar to those obtained via the REML estimator.

OLS results, obtained without specifying a different intercept for each landfill, are presented in Table 3 as possible benchmarks. OLS estimates present the same magnitude of the fixed effects of Table 2, with relatively small differences, confirming the sign of each coefficient. The models also share a satisfactory goodness of fit in terms of adjusted R^2^ (0.76 for model I and 0.79 for model II). Moreover, the absence of random intercepts seems to discharge the variability of the model in the presence of a post-operative fund and on the interaction between years after closure and presence of the fund. These two variables are statistically significant considering a nominal level of 0.01. For this reason, the estimate associated with the presence of a post-operative fund is 0.73 (Model I) or 0.75 (Model II); there is a slight decrease in costs for each additional year after closure in the presence of a post-operative fund.

**Table 3:** Results of OLS models

	*Model I* (*N* = 188)		*Model II* (*N* = 180)	
Variable	Estimate(SE)	t-test (signific.)	Estimate (SE)	t-test (signific.)
*Intercept*	5.841 (0.761)	***	5.077 (0.744)	***
*log_leachate*	0.428 (0.044)	***	0.443 (0.042)	***
*log_volume*	0.279 (0.071)	***	0.338 (0.069)	***
*Quantity_of_waste*	−0.001 (0.007)		0.007 (0.007)	
*Urban_waste*	−0.472 (0.249)	°	−0.671 (0.245)	**
*Years_aft_closure*	−0.027 (0.011)	*	−0.025 (0.010)	*
*Years_of_activity*	0.000 (0.006)		0.001 (0.006)	
*Management: Public*	−0.529 (0.135)	***	−0.533 (0.127)	***
*Property: Public*	0.174 (0.135)		0.222 (0.113)	°
*Connection to sewer*	−0.761 (0.138)	***	−0.768 (0.136)	***
*Post_op*	0.732 (0.233)	**	0.751 (0.229)	**
*Yrs_aft_clos∧Post_op*	−0.036 (0.013)	**	−0.039 (0.013)	**
Adjusted R^2^	0.761		0.789	

Significance symbols: *p*-value < 0.1 (°); *p*-value < 0.05 (*); *p*-value < 0.01 (**); *p*-value < 0.001 (***)

**Fig. 1:**
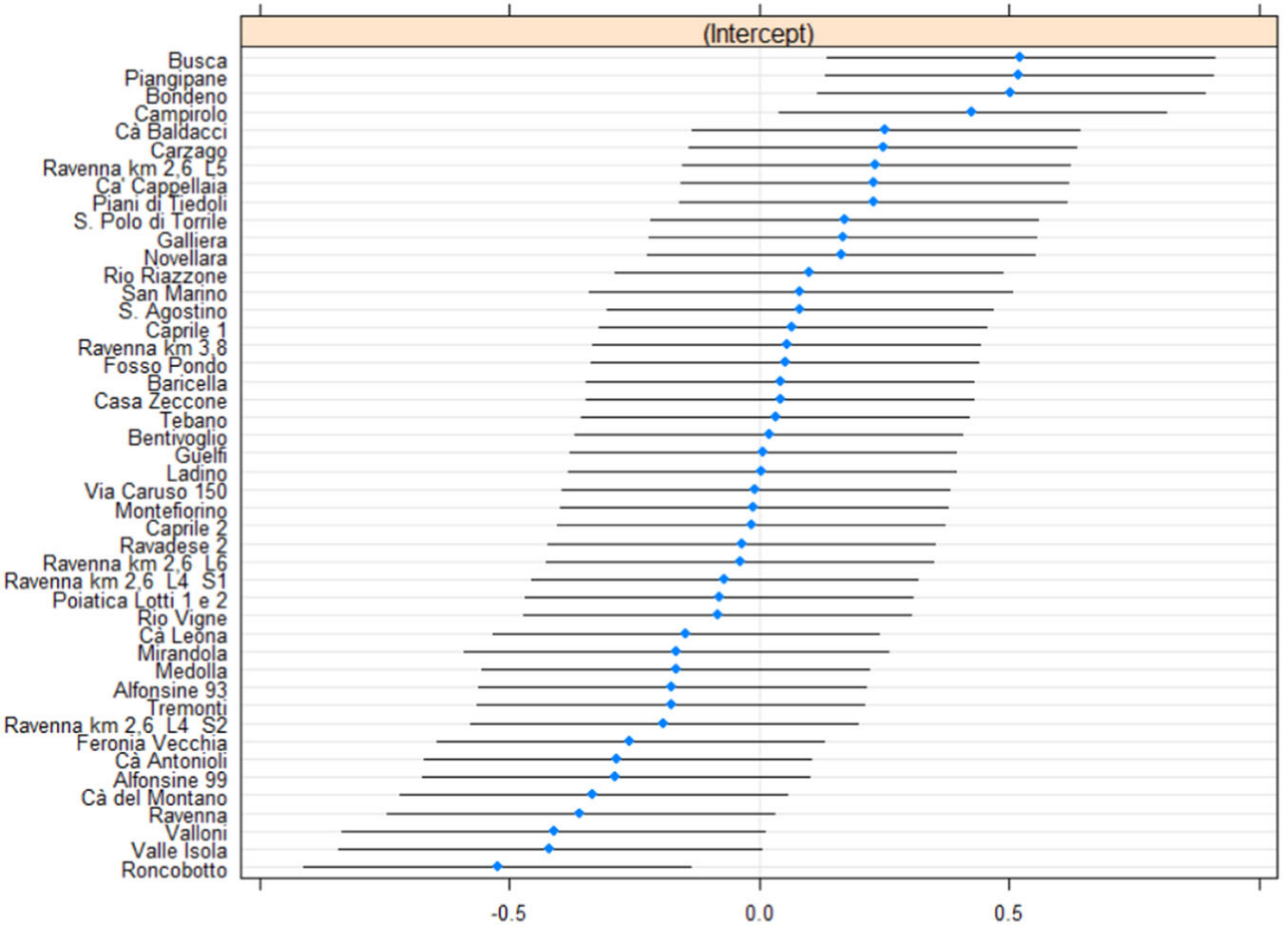
Random effect coefficients for Model II

For an in-depth analysis of the results of the random effect models, coefficients for the random intercepts of Model II and their confidence intervals at 95% are plotted in Fig. 1. Intervals that do not contain 0 indicate the presence of significant random intercepts. The landfills of Piangipane, Bondeno, and Busca present a highly positive random effect coefficient; while the first two share an increase in the costs over the considered period, the latter is the landfill with the highest logarithmic cost (on average) with respect to the whole sample. Indeed, Campirolo (the fourth landfill in the ranking of Fig. 1) exhibits one of the greater standard deviations for the costs over the considered period: the logarithm of costs decreased from 14.2 (2015) to lower than 11.5 (over three consecutive years). Conversely, the landfill of Roncobotto has a highly negative coefficient, and it is also the landfill with the lowest logarithmic cost (on average).

In conclusion, apart from the positive impact on the cost due to produced leachate and volume, closed landfills with public management and sewers and without a post-operative fund are more cost-efficient than others in the Emilia-Romagna region. The “seniority” of a landfill (after closure) is also a statistically significant variable but its magnitude in terms of impact on the cost is more negligible.

## Discussion

In this paper, we analyzed the comparative efficiency in the post-closure management of landfills between public and private sector operators. Concerning management type, our data showed that public landfills are more efficient than privately managed ones. This result casts doubt on the assumptions regarding the performance of private operators compared with public ones (Bel and Warner, 2008b; Peloza and Shang, 2011). Given the results, the supremacy of outsourcing is not supported (Peloza and Shang, 2011). Unlike Jacobsen et al. (2013), who highlighted that private management is more efficient in comparison with public management, our results found that evidence of cost saving in private management is ambiguous and not supported by empirical analysis (Bel and Costas, 2006; Bel and Warner, 2008b). This confirmed our assumptions, based on agency theory, that the private operator may not be efficient due to agency costs and/or opportunistic behavior and the non-alignment between the interests of the actors involved (Eisenhardt, 1989). Indeed, the absence of incentives and a formal control structure probably did not help align the interests of the principal agent (in this case ATERSIR) and the private partner and avoid opportunistic behavior. This is particularly relevant in post-closure management, as a contract may last 30 years or more, while the public maintains both the economic and environmental costs. This situation has implications in terms of the waste tariff that citizens pay as the inefficiencies are reversed in the tariff.

As highlighted by our assumptions based on stewardship theory, public operators may perform better than private ones. In this sense, public managers have probably been able to successfully compete with private ones (Boyne, 2002). In addition, public mangers could have maintained the public sector ethos and values at the base of the stewardship relationship with the general public and society (Bracci et al., 2021). The introduction of managerial culture in the public sector was the earliest goal of NPM. Our results open the debate on the compensation mechanism of full-cost recovery for waste management, which does not incentivize efficiency and gives margins of opportunism for the private operator. In this sense, there is a need to define good organizational structure to reduce agency cost and avoid rent-seeking behavior (Asenova and Beck, 2010; Jiang et al., 2021). Appropriate management control, governance, and regulatory structures, in this case, could be relevant (Appuhami et al., 2011) to reduce risk and increase efficiency (Langfield-Smith and Smith, 2003).

Other variables were also tested, confirming in most instances previous results. Predictably, a significant correlation between leachate and cost emerged from a simple regression model. These results confirmed the importance of cost deriving from leachate treatment for the performance of landfills (Camba et al., 2014). Another result that aligned with previous findings related to determining the total cost of landfill post-closure management is the connection to the sewage system (Berge et al. 2009). Our results confirmed that a connection to the sewage system reduces cost.

## Final Remarks and Limitations

This paper is, to the best of our knowledge, one of the first focusing specifically on how management types impact post-closure landfill efficiency. Old landfills are here to stay with potential future economic and environmental impacts. The results shed light on one of the basic tenets of NPM, namely that private operators are more efficient than public ones. Value for money (VFM) through privatization and/or liberalization, which is the basis for outsourcing public services, is not verified. Instead, value for money is linked to efficiency and effectiveness in delivering public services. Our study showed that through privatization, VFM is not proven from a cost efficiency point of view. The results reflect how the introduction of compulsory competitive tendering, to overcome the limits of public sector bureaucracy, did not lead to the expected results, or perhaps the benefits of externalization are lost over time. On the contrary, the results showed that public management is more efficient, contradicting the NPM reforms still in place.

These findings have implications for policy in relation to the primacy of private management in providing efficient public services. We have shown that keeping public management is more efficient than privatization. This probably explains the approach of some countries to re-nationalizing certain public services, including waste management services. Our results are important considering the consequences of issues related to COVID-19 in terms of the efficiency and long-term financial sustainability of public services. However, it is necessary to develop an appropriate organizational structure of regulation in terms of value for money to achieve performance goals and reduce agency cost. Otherwise, outsourcing, considering our results, is only a means to create rent-seeking opportunities for the private sector and to overcome limits or resource scarcity in the public sector. Another implication of the study is that efficiency is not equivalent to private management. Pursuing NPM and privatization reforms, which are associated with certain pre-conceived preferences in terms of the relationship between the type of management and efficiency, may not always be correct.

This research is not without its limitations, including the risk of errors in data registration from the landfill reports and the possibly “strict” assumption of the proposed linear mixed models. Furthermore, despite the model fitting, which could be considered quite satisfactory for different model specifications, other relevant variables were possibly excluded due to availability issues and missing data in the examined case, especially regarding waste composition. These problems were likely due to the age of the closed landfills and the need to improve their accountability. In further research, variables related to waste composition (e.g., the percentage of separate waste types) or the quantity of urban solid waste could be used in the proposed cost models.

## Appendix

Appendix. Results of PLM model

**Table:**

	*Model I (N* = *188)*		*Model II (N* = *180)*	
Fixed Effects	Estimate (SE)	z-test (signific.)	Estimate (SE)	z-test (signific.)
*Intercept*	6.746 (1.114)	***	5.255 (1.042)	***
*log_leachate*	0.484 (0.057)	***	0.492 (0.053)	***
*log_volume*	0.180 (0.101)	°	0.301 (0.094)	**
*Quantity_of_waste*	0.003 (0.009)		−0.005 (0.008)	
*Urban_waste*	−0.368 (0.346)		−0.658 (0.327)	*
*years_aft_closure*	−0.033 (0.015)	*	−0.027 (0.013)	*
*years_of_activity*	−0.005 (0.009)		−0.003 (0.008)	
*Management:Public*	−0.466 (0.202)	*	−0.482 (0.177)	**
*Property:Public*	0.108 (0.183)		0.201 (0.161)	
*Connection to sewer*	−0.706 (0.190)	***	−0.723 (0.180)	***
*Post_op*	0.494 (0.290)	°	0.550 (0.279)	*
*yrs_aft_clos∧Post_op*	−0.021 (0.017)		−0.027 (0.016)	°
Random Effects				
*Landfills*	0.219 (0.468)		0.219 (0.468)	
*Residuals*	0.161 (0.401)		0.110 (0.332)	
*Adjusted R*^*2*^	0.768		0.657	

Significance symbols: *p* value < 0.1(°); *p* value < 0.05(*); *p* value < 0.01 (**); *p* value < 0.001(***)

## Footnotes

1 https://www.arpae.it/it/temi-ambientali/rifiuti/impianti-rifiuti/discarica-rifiuti-non-pericolosi.

## References

[CR1] Alfiero S, Elba F, Esposito A, Resce G (2017). The impact of environmental factors on the measurement of managerial efficiency in the Italian waste management sector: Framework and empirical evidence. Int J Public Adm.

[CR2] Appuhami R, Perera S, Perera H (2011). Management controls in public-private partnerships: An analytical framework. Aust Account Rev.

[CR3] Asenova D, Beck M (2010). Crucial silences: When accountability met PFI and finance capital. Crit Perspect Account.

[CR4] Bates D, Mächler M, Bolker B, Walker S (2015). Fitting linear mixed-effects models using lme4. J Stat Softw.

[CR5] Behrooznia L, Sharifi M, Alimardani R, Mousavi-Avval SH (2018). Sustainability analysis of landfilling and composting-landfilling for municipal solid waste management in the north of Iran. J Clean Prod.

[CR6] Bel G, Costas A (2006). Do public sector reforms get rusty? Local privatization in Spain. J Policy Reform.

[CR7] Bel G, Warner M (2008). Guest editorial: Challenging issues in local privatization. Environ Plan. C Gov. Policy.

[CR8] Bel G, Warner M (2008). Does privatization of solid waste and water services reduce costs? A review of empirical studies. Resour Conserv Recycl.

[CR9] Benito-López B, Moreno-Enguix M, del R, Solana-Ibañez J (2011). Determinants of efficiency in the provision of municipal street-cleaning and refuse collection services. Waste Manag.

[CR10] Benito B, Bastida F, Garcia JA (2010). Explaining differences in efficiency: An application to Spanish municipalities. Appl Econ.

[CR11] Berenyi EB, Stevens BJ (2013). Privatization local work? Study of the delivery of eight services. State Local Gov Rev.

[CR12] Berge ND, Reinhart DR, Batarseh ES (2009). An assessment of bioreactor landfill costs and benefits. Waste Manag.

[CR13] Bilgili MS, Demir A, Özkaya B (2006). Quality and quantity of leachate in aerobic pilot-scale landfills. Environ Manage.

[CR14] Boyne GA (2002). Public and private management: What’s the difference?. J Manag Stud.

[CR15] Bracci E, Saliterer I, Sicilia M, Steccolini I (2021). Accounting for (public) value(s): reconsidering publicness in accounting research and practice. Accounting. Audit Account J.

[CR16] Brennan RB, Healy MG, Morrison L (2016). Management of landfill leachate: The legacy of European Union Directives. Waste Manag.

[CR17] Camba A, González-García S, Bala A (2014). Modeling the leachate flow and aggregated emissions from municipal waste landfills under life cycle thinking in the Oceanic region of the Iberian Peninsula. J Clean Prod.

[CR18] Croissant Y, Millo G (2008) Panel Data Econometrics in R: The PLM Package. J Stat Softw 27: 10.18637/jss.v027.i02

[CR19] Damgaard A, Manfredi S, Merrild H (2011). LCA and economic evaluation of landfill leachate and gas technologies. Waste Manag.

[CR20] Danthurebandara M, Van Passel S, Machiels L, Van Acker K (2015). Valorization of thermal treatment residues in Enhanced Landfill Mining: Environmental and economic evaluation. J Clean Prod.

[CR21] Davis JH, Schoorman FD, Donaldson L (1997). Toward a Stewardship Theory Of Management. 105465/amr19979707180258 22:20–47. 10.5465/AMR.1997.9707180258

[CR22] Di Foggia G, Beccarello M (2020). Drivers of municipal solid waste management cost based on cost models inherent to sorted and unsorted waste. Waste Manag.

[CR23] Dijkgraaf E, Gradus R (2007). Collusion in the Dutch waste collection market. Local Gov Stud.

[CR24] Dijkgraaf E, Gradus RHJM (2008). Institutional developments in the Dutch Waste-Collection market. Environ Plan C Gov Policy.

[CR25] Eisenhardt KM (1989). Agency Theory: An assessment and review. Acad Manag Rev.

[CR26] Fernández-Aracil P, Ortuño-Padilla A, Melgarejo-Moreno J (2018). Factors related to municipal costs of waste collection service in Spain. J Clean Prod.

[CR27] Ferronato N, Rada EC, Gorritty Portillo MA (2019). Introduction of the circular economy within developing regions: A comparative analysis of advantages and opportunities for waste valorization. J Environ Manage.

[CR28] Foss P (1995) Economic Approaches to Organizations and Institutions:… Dartmouth Publishing Company

[CR29] Franco DGdeB, Steiner MTA, Assef FM (2021). Optimization in waste landfilling partitioning in Paraná State, Brazil. J Clean Prod.

[CR30] Greco G, Allegrini M, Del Lungo C (2015). Drivers of solid waste collection costs. Empirical evidence from Italy. J Clean Prod.

[CR31] Greco G, Cenciarelli VG, Allegrini M (2018). Tourism’s impacts on the costs of municipal solid waste collection: Evidence from Italy. J Clean Prod.

[CR32] Grimsey D, Lewis MK (2005). Are public private partnerships value for money?: Evaluating alternative approaches and comparing academic and practitioner views. Account Forum.

[CR33] Guerrini A, Carvalho P, Romano G (2017). Assessing efficiency drivers in municipal solid waste collection services through a non-parametric method. J Clean Prod.

[CR34] Hefetz A, Warner M (2007). Beyond the market versus planning dichotomy: Understanding privatisation and its reverse in US cities. Local Gov Stud.

[CR35] Honma S, Hu JL (2021) Cost efficiency of recycling and waste disposal in Japan. J Clean Prod 284: 10.1016/j.jclepro.2020.125274

[CR36] Hood C (1991). A public management for all seasons. Public Adm.

[CR37] Hood C (1995). The “new” public management in the 1980s: variations on a theme. Accounting. Organ Soc.

[CR38] Jacobsen R, Buysse J, Gellynck X (2013). Cost comparison between private and public collection of residual household waste: Multiple case studies in the Flemish region of Belgium. Waste Manag.

[CR39] Jensen MC, Meckling WH (1976). Theory of the firm: Managerial behavior, agency costs and ownership structure. J Financ Econ.

[CR40] Jiang X-F, Zhao C-X, Ma J-J (2021). Is enterprise environmental protection investment responsibility or rent-seeking? Chinese evidence. Environ Dev Econ.

[CR41] Jiangying L, Dimin X, Youcai Z (2004). Long-term monitoring and prediction for settlement and composition of refuse in Shanghai Laogang Municipal Landfill. Environ Manage.

[CR42] Jones PT, Geysen D, Tielemans Y (2013). Enhanced Landfill Mining in view of multiple resource recovery: A critical review. J Clean Prod.

[CR43] Kale C, Gökçek M (2020). A techno-economic assessment of landfill gas emissions and energy recovery potential of different landfill areas in Turkey. J Clean Prod.

[CR44] Kelly RM (1998). An inclusive democratic polity, representative bureaucracies, and the new public management. Public Adm Rev.

[CR45] Kim KD, Lee EJ (2005). Potential tree species for use in the restoration of unsanitary landfills. Environ Manage.

[CR46] Kuznetsova A, Brockhoff PB, Christensen RHB (2017) lmerTest Package: Tests in Linear Mixed Effects Models. J Stat Softw 82:. 10.18637/jss.v082.i13

[CR47] Laner D, Crest M, Scharff H (2012). A review of approaches for the long-term management of municipal solid waste landfills. Waste Manag.

[CR48] Langfield-Smith K, Smith D (2003). Management control systems and trust in outsourcing relationships. Manag Account Res.

[CR49] Lee GF, Jones-Lee A (2004). Superfund site remediation by landfilling—overview of landfill design, operation, closure, and postclosure issues. Remediat J.

[CR50] Lombrano A (2009). Cost efficiency in the management of solid urban waste. Resour Conserv Recycl.

[CR51] Marques RC, Simões P (2008). Does the sunshine regulatory approach work. Resour Conserv Recycl.

[CR52] Massoud M, El-Fadel M (2002). Public–private partnerships for solid waste management services. Environ Manag.

[CR53] Mazzanti M, Montini A, Nicolli F (2010) Embedding landfill diversion in economic, geographical and policy settings. 101080/00036840903559612 43:3299–3311. 10.1080/00036840903559612

[CR54] Nai C, Tang M, Liu Y (2021). Potentially contamination and health risk to shallow groundwater caused by closed industrial solid waste landfills: Site reclamation evaluation strategies. J Clean Prod.

[CR55] Neto RO, Petter CO, Cortina JL (2009). Report: The current situation of sanitary landfills in Brazil and the importance of the application of economic models. Waste Manag Res.

[CR56] Nevrlý V, Šomplák R, Putna O, Pavlas M (2019). Location of mixed municipal waste treatment facilities: Cost of reducing greenhouse gas emissions. J Clean Prod.

[CR57] Nguyen HG, Nguyen DT, Nghiem HT (2021). Current management condition and waste composition characteristics of construction and demolition waste landfills in Hanoi of Vietnam. Sustainability.

[CR58] Niskanen A, Värri H, Havukainen J (2013). Enhancing landfill gas recovery. J Clean Prod.

[CR59] O’Flynn J (2007). From new public management to public value: Paradigmatic change and managerial implications. Aust J Public Adm.

[CR60] Pandey LMS, Shukla SK (2019). An insight into waste management in Australia with a focus on landfill technology and liner leak detection. J Clean Prod.

[CR61] Panzone L, Ulph A, Areal F, Grippo V (2021). A ridge regression approach to estimate the relationship between landfill taxation and waste collection and disposal in England. Waste Manag.

[CR62] Peloza J, Shang J (2011). How can corporate social responsibility activities create value for stakeholders? A systematic review. J Acad Mark Sci.

[CR63] Petersen T (1995) The Principal-Agent Relationship in Organisations. In: Economic approaches to organizations and institutions. Dartmouth

[CR71] R Core Team (2016) R: A Language and Environment for Statistical Computing, R Foundation for Statistical Computing, Vienna. Available at: www.R-project.org/

[CR64] Schillemans T, Bjurstrøm KH (2020). Trust and verification: balancing agency and stewardship theory in the governance of agencies. Int Public Manag J.

[CR65] Simões P, De Witte K, Marques RC (2010). Regulatory structures and operational environment in the Portuguese waste sector. Waste Manag.

[CR66] Swamy PAVB, Arora SS (1972). The exact finite sample properties of the estimators of coefficients in the error components regression models. Econometrica.

[CR67] Wang X, Mikulčić H, Dai G (2021). Decrease of high-carbon-ash landfilling by its Co-firing inside a cement calciner. J Clean Prod.

[CR68] Weng YC, Fujiwara T, Houng HJ (2015). Management of landfill reclamation with regard to biodiversity preservation, global warming mitigation and landfill mining: Experiences from the Asia-Pacific region. J Clean Prod.

[CR69] Yang Y, Jiang YH, lian XY (2016). Risk-based prioritization method for the classification of groundwater pollution from hazardous waste landfills. Environ Manage.

[CR70] Yarrow G, King M, Mairesse J, Melitz J (1986). Privatization in theory and practice. Econ Policy.

